# Industry-compatible silicon spin-qubit unit cells exceeding 99% fidelity

**DOI:** 10.1038/s41586-025-09531-9

**Published:** 2025-09-24

**Authors:** Paul Steinacker, Nard Dumoulin Stuyck, Wee Han Lim, Tuomo Tanttu, MengKe Feng, Santiago Serrano, Andreas Nickl, Marco Candido, Jesus D. Cifuentes, Ensar Vahapoglu, Samuel K. Bartee, Fay E. Hudson, Kok Wai Chan, Stefan Kubicek, Julien Jussot, Yann Canvel, Sofie Beyne, Yosuke Shimura, Roger Loo, Clement Godfrin, Bart Raes, Sylvain Baudot, Danny Wan, Arne Laucht, Chih Hwan Yang, Andre Saraiva, Christopher C. Escott, Kristiaan De Greve, Andrew S. Dzurak

**Affiliations:** 1 School of Electrical Engineering and Telecommunications, University of New South Wales, Sydney, New South Wales Australia; 2 Diraq, Sydney, New South Wales Australia; 3 Imec, Leuven, Belgium; 4 Department of Solid-State Sciences, Ghent University, Ghent, Belgium; 5 Department of Electrical Engineering (ESAT-MNS), KU Leuven, Leuven, Belgium

**Keywords:** Qubits, Electronic devices, Electrical and electronic engineering, Computational nanotechnology, Quantum dots

## Abstract

Among the many types of qubit presently being investigated for a future quantum computer, silicon spin qubits with millions of qubits on a single chip are uniquely positioned to enable quantum computing. However, it has not been clear whether the outstanding high-fidelity operations and long coherence times shown by silicon spin qubits fabricated in academic settings^1–8^ can be reliably reproduced when the qubits are manufactured in a semiconductor foundry^9–11^. Here we show precise qubit operation of silicon two-qubit devices made with standard semiconductor tooling in a 300-mm foundry environment. Of the key metrics, single- and two-qubit control fidelities exceed 99% for all four devices, and the state preparation and measurement fidelities reach up to 99.9%, as evidenced by gate set tomography. We report spin lifetime and coherence up to *T*_1_ = 9.5 s, ${T}_{2}^{* }=40.6\,{\rm{\mu }}{\rm{s}}$ and ${T}_{2}^{{\rm{Hahn}}}=1.9\,{\rm{ms}}$. We determine that residual nuclear spin-carrying isotopes contribute substantially to operational errors, identifying further isotopic purification as a clear pathway to even higher performance.

## Abstract (web summary)

Two-qubit operations exceeding 99% fidelity have been demonstrated by silicon devices made with standard semiconductor tooling in a 300-mm foundry environment.

## Main

The early academic successes of silicon spin-qubit technology^1–8^ created enthusiasm for the idea that these qubits could be fabricated in industrial 300-mm manufacturing lines^9–12^. Potential advantages include the ability to leverage advanced fabrication processes, to integrate qubits with modern electronics and to benefit from the existing capabilities of mass manufacturing at low costs^12^. Over the past decade, there have been a number of proposals for potential architectures that could be used to realize a scalable quantum computer based on silicon spin qubits^13–17^.

However, recent attempts to fabricate spins-in-silicon quantum dots using industrial-scale fabrication has raised awareness about the material challenges that must be faced. For example, it is crucial to reduce the charge noise and static disorder arising due to defects and traps at interfaces and oxides^18–20^. High-fidelity single-qubit operations can be performed with minimal impact from charge noise because the electron spin couples to the noisy electric fields only through its weak spin–orbit effects^21–25^. By contrast, other operations, including qubit initialization, read-out and exchange-based two-qubit gates, are more sensitive to the charge noise present in conventional metal–oxide–semiconductor gate stacks^19,24^.

This sensitivity has motivated the search for ways of mitigating the effects of charge noise, including moving spin states away from the interface to quantum wells^26,27^. However, from a scaling perspective, it is desirable to reconcile as much as possible conventional complementary metal–oxide–semiconductor (CMOS) processes with qubit fabrication to leverage the mature CMOS industry to the fullest extent. Moreover, the formation of quantum dots against thin oxides has several benefits, such as a large electric field that leads to large valley splitting^18,28,29^ and efficient electrostatic coupling between the gate voltage bias and the channel, allowing for well-isolated states, efficient exchange coupling control^29^ and fast gate-based read-out^30^.

In this work, we study four two-qubit devices fabricated using a long-established CMOS geometry, namely a planar metal–oxide–semiconductor with polysilicon gates^31^. We demonstrate for all devices all basic qubit operations, including one- and two-qubit gates, initialization and read-out, with error rates approaching and surpassing the fault-tolerance threshold for the widely studied surface error correction code^32^. We obtain these error rates using state-of-the-art gate set tomography (GST) tools^33,34^, allowing us to pinpoint effects such as crosstalk and the breakdown between stochastic and coherent errors. Our studies indicate unprecedented reproducible low error levels while also identifying clear pathways to further reducing errors.

## Device operation

**Fig. 1:**
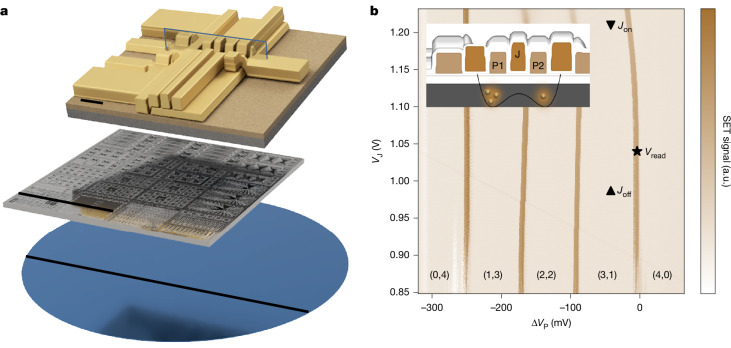
Two-qubit device and operation points. **a**, Schematic of a Diraq two-qubit device fabricated on a 300-mm wafer, showing the full wafer, single die and single device level. **b**, Charge stability diagram of device A as a function of plunger (P1 and P2) voltage detuning Δ*V*_P_ = Δ*V*_P1_ = −Δ*V*_P2_ and exchange gate (J) voltage *V*_J_, showing four isolated electrons in the double-dot system. Devices B–D were also measured in the (3,1) charge configuration and showed analogous charge stability diagrams. Voltage points for single-qubit operation (*J*_off_ < 1 kHz), two-qubit operation (*J*_on_ ≈ 1 MHz) and read-out are shown as a triangle, inverted triangle and star, respectively. The faint charge transition starting from *V*_J_ = 1.05 V is very weakly coupled to the quantum dot and probably corresponds to charge movement outside the active dot confinement area. In device D, we observed similar charge movements outside the active dot region; devices B and C showed none. Inset, Cross section of the double-quantum-dot device indicating the number of electrons under the plunger gates (P1 and P2), exchange gate (J) and double potential well. Scale bars, 100 nm (**a**, top), 10 mm (**a**, middle), 300 mm (**a**, bottom). a.u., arbitrary units.

The qubit devices were designed by Diraq and fabricated at imec in a 300-mm spin-qubit process flow that is described in Methods. The resulting wafer consists of several devices covering a range of feature sizes and configurations. We chose the design that we estimated would have optimal device parameters, and we tested four of these devices (denoted A–D). The design consists of a double quantum dot and a nearby single-electron transistor (SET) for spin read-out^1^ (Fig. 1a).

**Extended Data Fig. 1:**
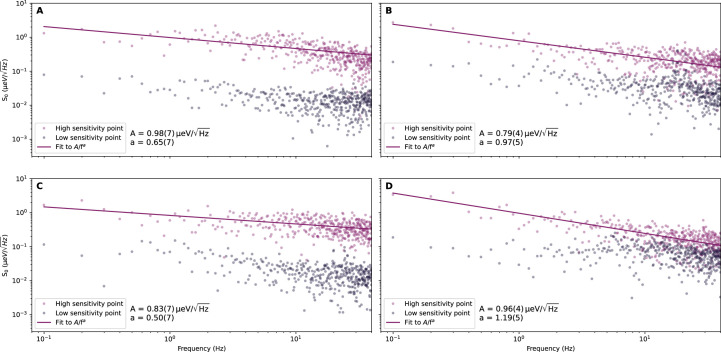
Single electron transistor charge noise spectrum. Noise spectrum at a high and a low-sensitivity point of a Coulomb oscillation at *B*_0_ = 0.662 − 0.7 T for devices A–D. Voltage noise spectrum is first calculated using a Fast Fourier transform on extracted SET current measurements divided by the slope of the Coulomb peak, and finally converted into a charge noise spectrum in μeV using the lever arm^58^. Charge noise at 1 Hz ranges between $0.79(4)-0.98(7)\,{\rm{\mu }}{\rm{e}}{\rm{V}}/\sqrt{{\rm{H}}{\rm{z}}}$. Solid line is a power law fit of the power spectral density with exponent ranging between *a* = 0.50(7) − 1.19(5).

We operated the devices in a ^3^He/^4^He dilution refrigerator with a base temperature of 10 mK in isolated mode, with four electrons in the double dot formed under the plunger gate electrodes (P1 and P2). A voltage applied to an interstitial exchange gate (J) controlled the tunnel coupling between the dots^1,35,36^. The electrons were loaded from a two-dimensional electron gas formed under the reservoir gate, which overlaps with an n^++^ implanted ohmic region. The Pauli spin blockade at the (3,1)–(4,0) charge transition was used for spin parity read-out and the signal was measured using the SET in d.c. mode (Fig. 1b). SET charge-noise measurements reveal a 1/*f*^*α*^ power spectral density with *α* between 0.50(7) and 1.19(5) and an amplitude of 0.79(4)–$0.98(7)\,{\rm{\mu }}{\rm{eV}}/\sqrt{{\rm{Hz}}}$ at 1 Hz (Extended Data Fig. 1), in line with previous low charge-noise results from devices fabricated using similar methods^19,21^.

**Fig. 2:**
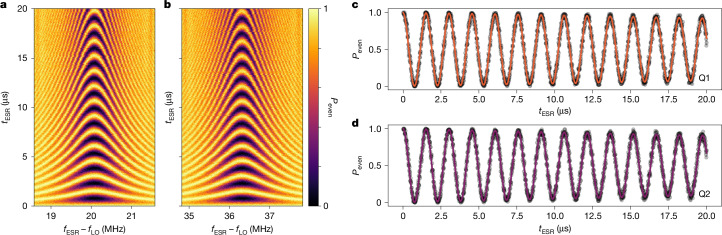
Single-qubit operation of device A. **a**,**b**, Rabi chevrons for qubits 1 (**a**) and 2 (**b**). Probability of reading out an even or blockaded spin state *P*_even_, when applying microwave signal at the frequency *f*_ESR_ – *f*_LO_ for a duration of *t*_ESR_. **c**,**d**, Coherent Rabi oscillations at *f*_L,Q*i*_ for qubits 1 (**c**) and 2 (**d**). Real-time feedback is implemented to counteract the Larmor frequency deviation^39^. The local oscillator frequency is set to *f*_LO_ = 18.610 GHz. The microwave power applied to qubit 1 is approximately 7% larger than that to qubit 2 resulting in matching Rabi frequencies of *f*_Rabi_ = 658.6(3) kHz.

A static magnetic field *B*_0_ in the range 0.662–0.7 T split the spin states to form the single-qubit states, which were manipulated with on-resonance microwave signals applied to the electron spin resonance antenna with frequency *f*_ESR_. Single-qubit X_π/2_ gates were formed with timed microwave pulses on resonance with the qubit Larmor frequencies *f*_L,Q1_ and *f*_L,Q2_ (Fig. 2), whereas Z_π/2_ gates were implemented as virtual gates through rotations of the reference frame^37^. The single-qubit metrics, including spin lifetime and Ramsey and Hahn coherence times, for the four devices are shown in Table 1.

**Table 1:** Device performance reproducibility

Device	*B*_0_ (mT)	Fidelity (%)					*T*_1_ (s)		${{\boldsymbol{T}}}_{{\bf{2}}}^{{\boldsymbol{* }}}$ (μs)		${{\boldsymbol{T}}}_{{\bf{2}}}^{{\bf{Hahn}}}$ (μs)	
		SPAM	XI	IX	II	CZ	Q1	Q2	Q1	Q2	Q1	Q2
A	666	99.95(8)	99.48(5)	99.55(4)	99.74(9)	99.37(5)	2.4(2)	6.3(6)	30.2(6)	29.1(6)	445(6)	803(6)
B	662	99.33(9)	99.47(6)	99.18(5)	99.89(9)	99.56(6)	0.9(2)	0.8(1)	30.5(2)	26.2(2)	811(24)	148(4)
C	700	99.97(22)	99.59(15)	99.51(14)	99.81(6)	99.04(16)	1.3(2)	1.9(2)	34.2(1.1)	32.5(2.3)	1,569(56)	1,044(52)
D	700	99.96(5)	99.23(3)	99.64(3)	99.62(6)	99.20(3)	1.8(2)	0.12(1)	21.5(4)	9.3(1)	357(15)	118(2)

**Extended Data Fig. 2:**
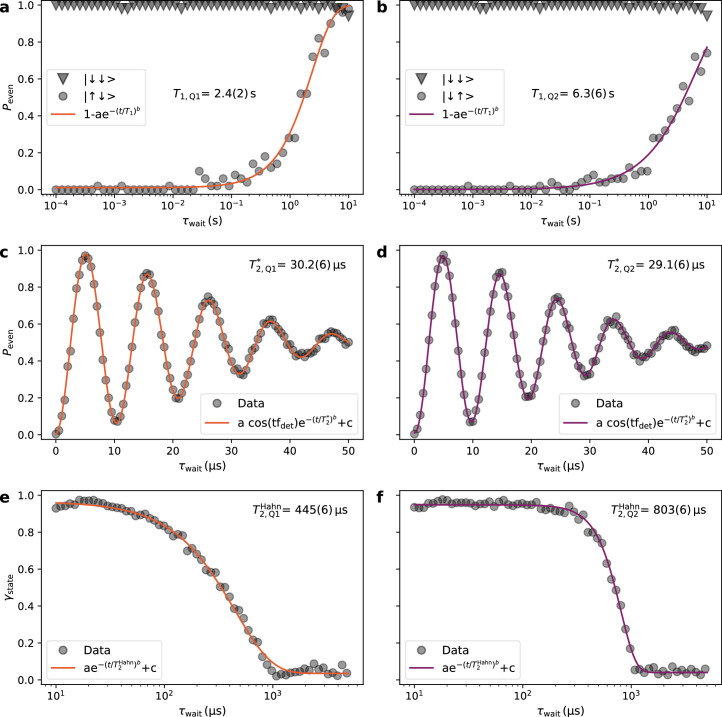
Single qubit metrics. **a**,**b**, Spin relaxation of qubit 1 (**a**) and 2 (**b**) spin up to the ground state $| \downarrow \downarrow \rangle$. Curves are fitted to an exponential decay resulting in a relaxation time of *T*_1,Q1_ = 2.4(2) s (a = 0.999, b = 0.847) and *T*_1,Q2_ = 6.3(6) s (a = 0.989, b = 1.183), respectively. For long wait times, the pure $| \downarrow \downarrow \rangle$ state decays to its thermal equilibrium following the Fermi-Dirac distribution at finite temperatures. **c**,**d**, Ramsey experiment for qubit 1 (**c**) and 2 (**d**) fitted to a sinusoidal exponential decay resulting in a coherence time of ${T}_{2,{\rm{Q}}1}^{* }=30.2(6)\,{\rm{\mu }}{\rm{s}}$ (a = −0.498, *f*_det_ = 95 kHz, b = 1.874, c = 0.498) and ${T}_{2,{\rm{Q}}2}^{* }=29.1(6)\,{\rm{\mu }}{\rm{s}}$ (a = −0.488, *f*_det_ = 102 kHz, b = 1.884, c = 0.499), respectively. The oscillation is induced by a phase shift corresponding to a 100 kHz detuning. Every data point is averaged for 1,000 repeats, each integrating the readout signal for *t*_int_ = 100 μs. We use real-time Larmor frequency feedback between repeats using the protocol described in ref. ^39^. **e**,**f**, Hahn echo experiment for qubit 1 (**e**) and 2 (**f**) fitted to an exponential decay resulting in a coherence time of ${T}_{2,{\rm{Q}}1}^{{\rm{Hahn}}}=445(6)\,{\rm{\mu }}{\rm{s}}$ (a = 0.931, b = 1.263, c = 0.035) and ${T}_{2,{\rm{Q}}2}^{{\rm{Hahn}}}=803(6)\,{\rm{\mu }}{\rm{s}}$ (a = 0.908, b = 3.125, c = 0.040), respectively. We measure all six single qubit projections and fit the state purity *γ*_state_. Error bars represent the 95% confidence level.

Operation fidelities of four nominally identical devices from the same 300-mm wafer benchmarked using GST. Spin lifetime and coherence metrics across devices are obtained following Extended Data Fig. 2. Errors are based on the 95% confidence level.

**Fig. 3:**
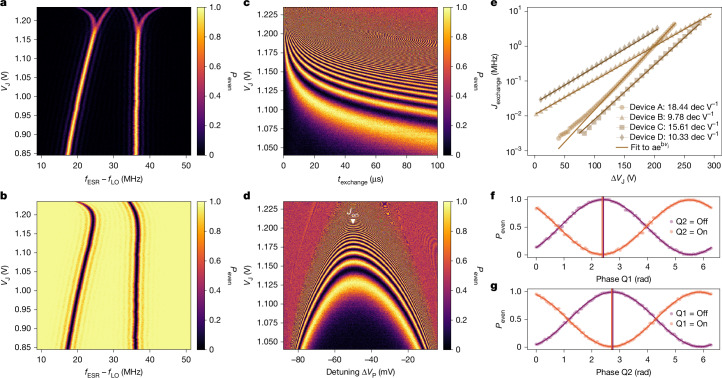
Two-qubit operation of device A. **a**,**b**, Probability of detecting an even spin state *P*_even_ after a microwave burst of fixed power and duration at different J gate voltages *V*_J_ when preparing a mixed odd state of $| \downarrow \uparrow \rangle$ and $| \uparrow \downarrow \rangle$ (**a**) and a pure even state $| \downarrow \downarrow \rangle$ (**b**). The power and duration of the microwave burst are roughly calibrated to a single-qubit π rotation. The local oscillator frequency is set to *f*_LO_ = 18.610 GHz. The mixed odd state is initialized with a *t*_ramp_ = 1 μs ramp at *V*_J_ = 0.96 V over the anti-crossing and the even state with heralded initialization based on a *t*_ramp_ = 1 μs ramp at *V*_J_ = 1.12 V. All experiments throughout the paper were conducted with an even state $| \downarrow \downarrow \rangle$ initialization, unless otherwise specified. **c**, Controlled phase (CZ) oscillation as a function of exchange time *t*_exchange_ and exchange gate voltage *V*_J_. We apply dynamical decoupling by a consecutive π rotation on both qubits to filter out other phase-accumulating effects, such as Stark shift. **d**, Decoupled exchange oscillation fingerprint for fixed exchange time *t*_exchange_ = 10 μs as a function of plunger voltage detuning Δ*V*_P_ and exchange gate voltage *V*_J_. The triangle indicates the exchange gate voltage used for the CZ gate. **e**, Exchange strength as a function of exchange gate voltage extracted from fitting exchange oscillations in **c**. The controllability is fitted assuming *J*_exchange_ ∝ *a* exp *bV*_J_, resulting in a range between *b* = 9.78 dec V^−1^ and 18.44 dec V^−1^ across devices A–D. The variation in exchange controllability across the devices is probably due to fabrication variability. **f**,**g**, Calibration of the CZ single-qubit phase correction by preparing the target spin in superposition, applying a CPHASE followed by a virtual phase rotation for qubits 1 (**f**) and 2 (**g**)^4,7^. Vertical lines indicate the phase values for which the spin state of Q1 (Q2) is flipped if Q2 (Q1) is in the on/spin-up state. The read-out probability is unscaled in all data.

**Extended Data Fig. 3:**
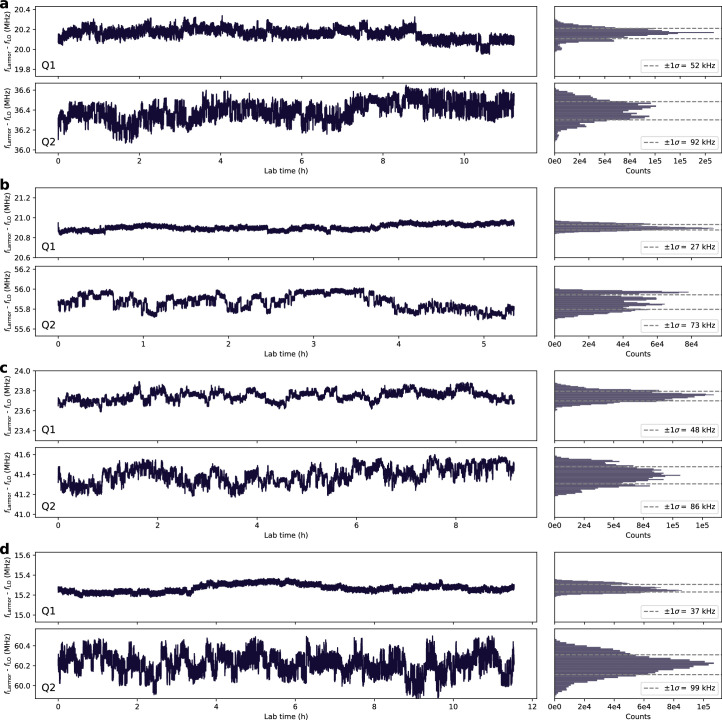
Single qubit Larmor frequency feedback. **a**–**d**, Single qubit Larmor frequency deviation of qubit 1 and 2 across devices A – D (**a**–**d**) as a function of lab time during a GST experiment nominally equivalent to the ones discussed in the main text (Fig. 4). Figures are plotted with the same *y*-axis range. The standard deviation of the frequency feedback of qubit 2 (73–99 kHz) is larger compared to qubit 1 (27–52 kHz) across all devices. The microwave local oscillator frequency is set to *f*_LO_ = 18.610−19.590 GHz (compare Table 1). Frequency feedback is implemented by following the protocol described in ref. ^39^.

Figure 3 details our implementation of two-qubit control. A voltage pulse, typically of duration *t*_CZ_ between 100 ns and 500 ns, was applied to the J gate to control the exchange interaction strength (Fig. 3a–c). We performed the two-qubit gate at the symmetric charge operation point to reduce the sensitivity to the detuning noise^38^ (Fig. 3d). We extracted from the fitted exchange frequencies in Fig. 3c an exchange controllability of 18.4 dec V^−1^ (devices A–D are compared in Fig. 3e). By combining a pulsed exchange interaction with single-qubit phase rotations, we were able to implement a controlled-*Z* (CZ) two-qubit gate^1^ (Fig. 3f,g). Using real-time feedback, we tracked and corrected the SET voltage operation point and the single-qubit Larmor and Rabi frequencies^39^ (see Extended Data Fig. 3 for details). We used a heralded initialization protocol to check if the qubit system was successfully initialized in the $| \downarrow \downarrow \rangle$ state, and we repeated the initialization if unsuccessful^7,40^.

## Two-qubit benchmarking

To precisely capture errors in qubit operation, we designed a GST experiment comprising the full gate set {II, XI, IX, ZI, IZ, CZ}, from which we constructed a list of ‘preparation’ and ‘measurement fiducials’ circuits. Combining these fiducials with ‘germ’ circuits up to length 16, we obtained a list of 12,263 unique sequences^34^. We chose GST as the benchmarking method because it yields the error taxonomy with specific error channels and is generally a higher bar for fidelities than the more commonly used interleaved randomized benchmarking, which often overestimates fidelities^8^.

We implemented an idle gate with a total idle time equal to the duration of a CZ gate. Single-qubit gates in the two-qubit context were achieved by rotating one qubit π/2 around the $\widehat{x}$ axes (XI and IX) and $\widehat{z}$ axes (ZI and IZ), while the other qubit was idling. The XI and IX gates were implemented by applying a microwave square pulse of around 400 ns, including a 20-ns smooth sine ramp, at *f*_L,Q1_ and *f*_L,Q2_ for qubits 1 and 2, respectively. The ZI and IZ gates were implemented by virtually rotating the reference frame for qubits 1 and 2, respectively. The entangling two-qubit CZ gate was implemented by applying a voltage pulse to the exchange gate J for a duration of *t*_CZ_.

**Fig. 4:**
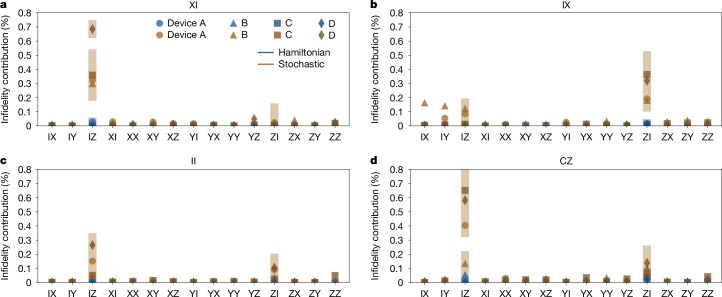
Two-qubit benchmarking using GST. **a**–**d**, Infidelity contributions of Hamiltonian and stochastic error generators across all four devices for the XI (**a**), IX (**b**), II (**c**) and CZ (**d**) gates from GST. Hamiltonian errors contribute to the infidelity in second order, whereas stochastic errors contribute in first order. Stochastic errors, particularly the idling ZI and IZ error generator components, dominate the infidelity by more than an order of magnitude. All gate fidelities across all devices are above 99%. Error bars represent the 95% confidence level. For visibility, only those of the IZ and ZI error generators are displayed.

**Extended Data Fig. 4:**
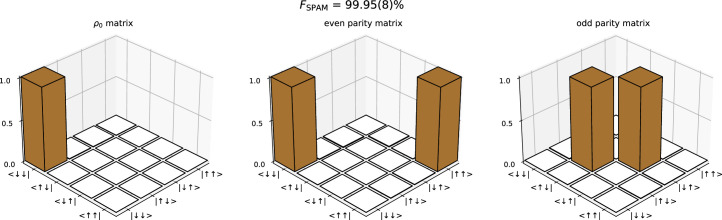
State preparation and measurement. GST-estimated SPAM operations, with the initial state represented as a density matrix and the terminating parity measurement as a POVM, yield a $| \downarrow \downarrow \rangle$ state initialization fidelity of *F*_init_ = 99.996(49)% and an even/odd parity measurement of *F*_meas_ = 99.959(59)%, respectively. The combined state preparation and measurement fidelity is calculated to *F*_SPAM_ = 99.95(8)%. Error bars represent the 95% confidence level.

Figure 4 breaks down the error generators and their contribution to the infidelity for each gate and device. Across all four devices operation accuracies are above 99%. For example, for device A, the gate fidelities are 99.45(5)%, 99.55(4)%, 99.95(4)%, 99.96(4)%, 99.74(9)% and 99.37(5)% for the XI, IX, ZI, IZ, II and CZ, respectively (see first row of Table 1). The combined state preparation and measurement (SPAM) fidelity even exceeds 99.9% with *F*_SPAM_ = 99.95(8)%, which is one of the highest reported values for industrially fabricated spin qubits (Extended Data Fig. 4). Single-qubit on-target gate fidelities *F*_X,Q1_ = 99.97(15)% and *F*_X,Q2_ = 99.81(11)% are comparable to previously reported values for devices made using the same fabrication processes^21^.

## Reproducibility

All four devices have fidelities of 99% or higher across all operations, with XI (IX), ZI (IZ) and CZ gates ranging between 99.23(3)% and 99.59(15)% (99.18(5)% and 99.64(3)%), between 99.95(5)% and 99.97(3)% (99.95(5)% and 99.97(3)%) and between 99.04(16)% and 99.56(6)% (Table 1). Furthermore, the stochastic IZ and ZI error generators dominate the infidelity contributions across all devices. In particular, the stochastic IZ component is larger for the CZ gate compared with the I gate, which is probably related to the (3,1) charge configuration used in all devices. In future work, a systematic study of the (1,3) charge configuration, as well as higher electron occupations such as (3,3) across several devices, would provide valuable insights into the physical origin of the observed error mechanisms. SPAM fidelities reproducibly exceed 99.9% for three out of the four devices (devices A, C and D). For device B, we achieved a SPAM fidelity of 99.33(9)%. We attribute this slightly reduced SPAM fidelity to signs of gate leakage between the SET barrier and the ohmic contacts. Similarly, device D shows leakage between the SET barrier and the plunger gate used to form qubit 2, which is the probable cause of the reduced spin relaxation and coherence time of qubit 2 (Table 1).

**Extended Data Fig. 5:**
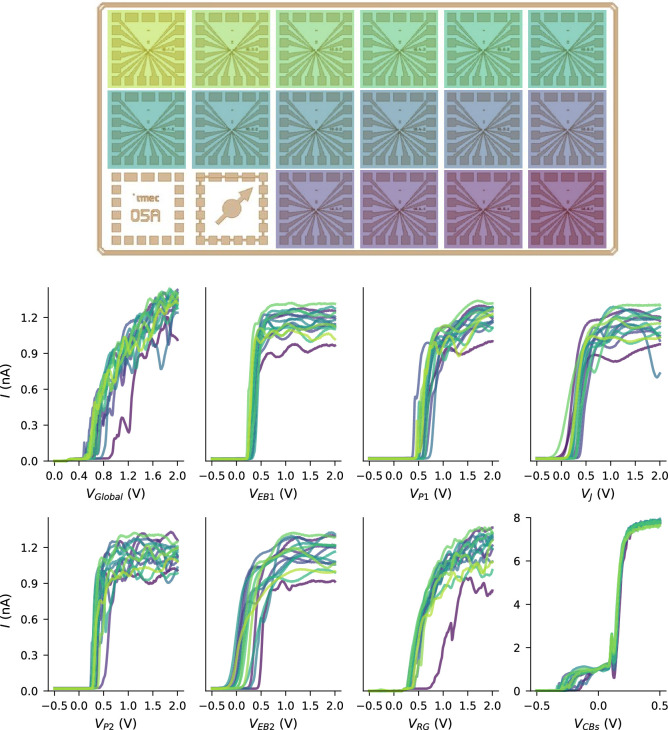
Global turn-on and gate pinch-off curves. Each plot shows I–V data for 16 quantum-dot devices (coloured curves) measured with a cryo-prober at around 1 K. One device with same design parameters as devices A–D, and 15 with different design parameters, all from the same 300 mm wafer. Different panels pertain to the gate electrodes (EB1, P1, J, P2, EB2, RG, CBs) or to all gates (top left).

Additionally, we performed d.c. cryo-probing measurements on a further 16 devices: one with the same design parameters as devices A–D and 15 with different design parameters, all from the same imec wafer. These measurements indicate excellent uniform electrostatic control over all device gate electrodes (Extended Data Fig. 5).

## Discussion

Device A exemplifies the high performance of qubits on the 300-mm wafer and can, therefore, be used as a case study to examine the sources of infidelity. In the following sections, we analyse the GST error contributions and their probable physical origins in device A, as well as its qubit performance as a function of magnetic field. Based on the consistency of the dominating errors reported in Fig. 4 and the similarity of the Larmor frequency dynamics in Extended Data Fig. 3, we expect this conclusion to hold for all four devices.

The ZI and IZ gates were implemented virtually and are, therefore, in principle error-free. However, the clock update on the field-programmable gate array took a finite time, leading to slight imperfections in these virtual operations due to idling errors. Hence, we attribute their infidelities, 0.05(4)% (0.04(4)%) for ZI (IZ), to a small time delay in the control hardware.

Most importantly, the largest contribution to infidelity for the XI, II and CZ gates is the stochastic IZ error generator, whereas the IX gate is limited by the stochastic ZI error generator, with the second highest contribution being the stochastic IZ error generator, followed by the stochastic IY error generator (Fig. 4). The stochastic dephasing error generator of the qubit undergoing a π/2 rotation (ZI for the XI gate and IZ for the IX gate) is significantly larger for the IX gate than for the XI gate. However, for the idle qubit (IZ for the XI gate and ZI for the IX gate), it is significantly larger for the XI gate than for the IX gate. Similarly, the infidelity contribution to the II gate from the stochastic dephasing error generator IZ (0.15%) is larger than for ZI (0.09%).

First, these error contributions could be explained by the coupling of ^29^Si nuclear spins to the electron spin state of qubit 2 (ref. ^41^). Qubit 1 also experiences quasi-static changes in the Larmor frequency, but these are of lower magnitude, despite the Stark shifts of qubit 1 being larger (Extended Data Fig. 3). This is another indication that hyperfine-coupled residual ^29^Si nuclear spins are the origin of that noise. As expected, these idling errors are roughly doubled (*t*_XI_ = *t*_IX_ = 400 ns) in comparison with the idling gate, which only takes around half the time. Comparing the II gate with the CZ gate, the stochastic ZI error generator remains constant (0.09%), but the IZ error generator more than doubles (0.40%), despite both gates having the same duration *t*_II_ = *t*_CZ_ = 212 ns. However, during CZ gate operation, the tunable exchange barrier is lowered to enable the Heisenberg exchange. This leads to a shift in the centre of the electron wavefunction, possibly moving qubit 2 closer to the ^29^Si nuclear spin, thus increasing the contact hyperfine interaction and the quasi-static noise in IZ.

**Extended Data Fig. 6:**
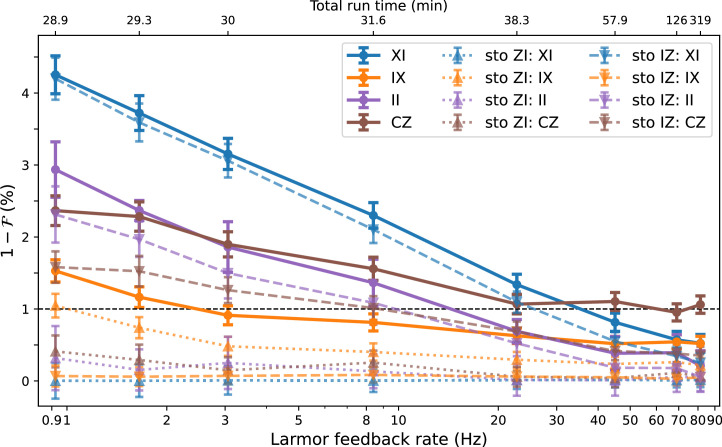
Gate infidelity as function of Larmor feedback rate. Gate infidelities $1-{\mathcal{F}}$ benchmarked using GST as a function of Larmor frequency feedback rate (the frequency at which the feedback is executed and the Larmor frequency updated) at *B*_0_ = 0.666 T. We estimate the precision to be ~5 kHz, based on the deviation from the induced 100 kHz oscillations in Ramsey experiments shown in Extended Data Fig. 2. The virtual ZI and IZ gates are independent of the Larmor frequency and omitted. Each GST experiment consists of 12,263 sequences repeated 100 times. The Larmor frequency feedback circuit is executed with increasing rate, i.e. after every 3000th, 1000th, 300th, 100th, 30th, 10th, 3rd, and single GST gate sequence. All gate infidelities decrease with increasing Larmor frequency feedback rate. The dominant infidelity contribution to the XI, II, and CZ gate is the stochastic IZ error generator, originating from idling of qubit 2. The IX gate infidelity is dominated by the respective ZI error generator, originating from idling of qubit 1. Hamiltonian ZI and IZ error generators, not plotted here, contribute about 0.4% to the infidelity of the CZ gate. Better calibration, as done in the main text (Fig. 4), removes the Hamiltonian errors almost entirely and brings the all gate infidelities below the commonly targeted 1% infidelity threshold represented by the black dashed line. Error bars represent the 95% confidence level.

Second, it might seem contradictory that the gate infidelity is dominated by noise in qubit 2, given that ${T}_{2,{\rm{Q}}2}^{{\rm{Hahn}}} > {T}_{2,{\rm{Q}}1}^{{\rm{Hahn}}}$ and ${T}_{2,{\rm{Q}}2}^{* }\approx {T}_{2,{\rm{Q}}1}^{* }$. However, the GST experiments are designed to amplify, not echo out, persistent noise sources. They are, therefore, heavily affected by slow noise, such as Larmor frequency changes arising from hyperfine-coupled nuclear spins. On the other hand, Hahn echo experiments are limited by fast charge noise. The Larmor frequency variation, tracked during the GST experiment using real-time feedback^39,40^, shows a higher standard deviation for the qubit 2 frequency variation compared with qubit 1 (Extended Data Fig. 3). Furthermore, the infidelities of the XI, IX, II and CZ gates decrease for higher Larmor feedback rates, with the main contribution again being the stochastic IZ error generator for the XI, II and CZ and the stochastic ZI error generators for the IX gate (Extended Data Fig. 6).

**Extended Data Fig. 7:**
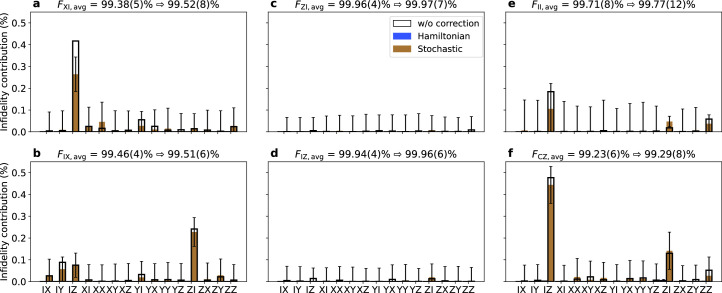
Post-selected 2-qubit benchmarking. Infidelity contribution of Hamiltonian and stochastic error generators of the full gate set XI (**a**), IX (**b**), ZI (**c**), IZ (**d**), II (**e**), and CZ (**f**) comparing the effect of removing gate sequences with Larmor frequency jumps > 50 kHz in post-selection at *B*_0_ = 0.666 T using GST. Hamiltonian errors contribute to the infidelity in second order, while stochastic errors contribute in first order. The ZI and IZ gates are implemented virtually and are independent of that post selection within error bars. The original (without post-selection) GST experiment consisted of 12,263 sequences, with Larmor frequency feedback after every 10th sequence, repeated 400 times. 171 repeats passed the post-selection for analysis. Error bars represent the 95% confidence level.

**Extended Data Fig. 8:**
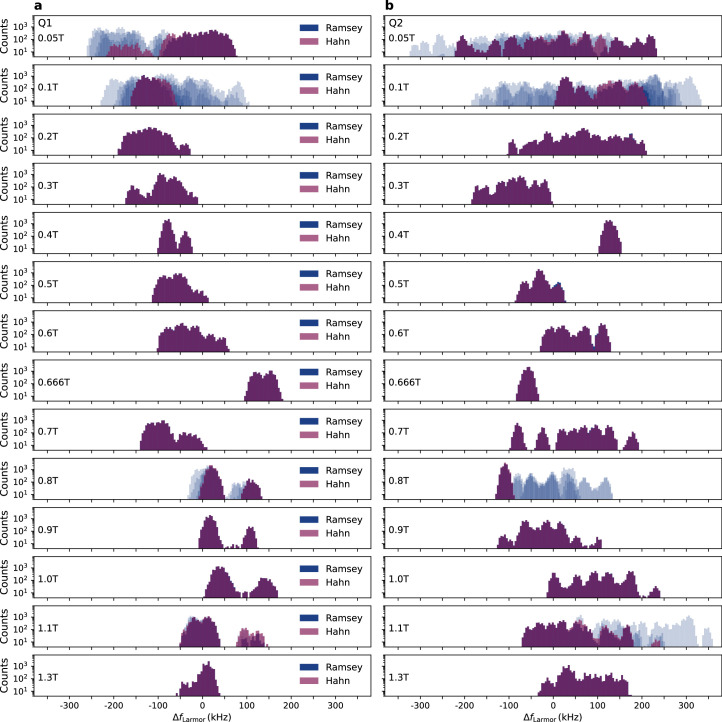
Larmor frequency feedback as function of *B*_0_. **a**,**b**, Larmor frequency feedback *σ*(Δ*f*_L_) during interleaved Ramsey and Hahn echo measurements for different magnetic field amplitudes *B*_0_ of qubit 1 and 2, respectively. Each Ramsey experiment is repeated at least 10,000 times, with Larmor frequency feedback before each repeat, corresponding to 10,000 data points and roughly 25 min. For magnetic field amplitudes of *B*_0_ = 0.05 T, 0.1 T, 0.8 T, and 1.1 T, 50,000, 100,000, 50,000, and 40,000 Ramsey repeats are measured and then truncated in post batched in chunks of 10,000 repeats displayed here shaded. Each Hahn experiment is repeated 2,000 times for all six state projections [+X,−X,+Y,−Y,+Z,−Z], with Larmor feedback between each repeat for each projection, corresponding to 12,000 data points and roughly 30 min. The Hahn echo experiment for *B*_0_ = 1.3 T is only repeated 500 times.

**Extended Data Fig. 9:**
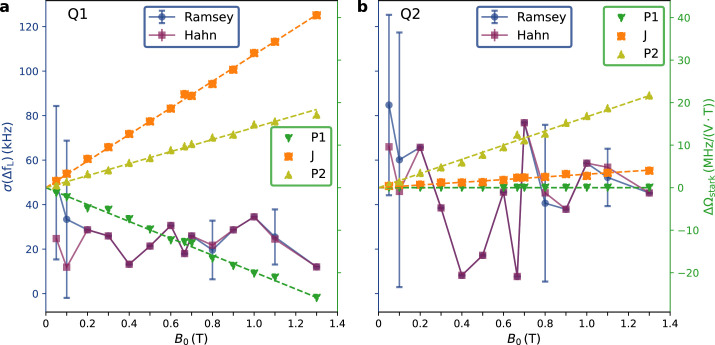
Larmor frequency and Stark shift as function of *B*_0_. **a**,**b**, Standard deviation of the Larmor frequency variation *σ*(Δ*f*_L_) obtained from real-time Larmor frequency feedback during interleaved Ramsey and Hahn echo measurements as a function of magnetic field amplitudes *B*_0_ of qubit 1 and 2, respectively. The second y-axis on the right shows the Stark shift Δ*Ω*_Stark_ of three gates P_1_, J, and P_2_ in the active region forming the double-dot system. Dashed lines follow a linear fit Δ*Ω*_Stark_ ⋅ *B*_0_, resulting in Δ*Ω*_Stark_(P1, J, P2) = (−19.9(4), 31.3(3), 14.2(4)) MHz/(V·T) and = (0, 3.2(2), 16.7(4)) MHz/(V·T) for qubit 1 and 2, respectively. Error bars for the standard deviation of the Larmor frequency variation are only extracted for magnetic field amplitudes of *B*_0_ = 0.05 T, 0.1 T, 0.8 T, and 1.1 T with 50,000, 100,000, 50,000, and 40,000 Ramsey repeats batched in chunks of 10,000, respectively. Error bars represent the 95% confidence level.

**Extended Data Fig. 10:**
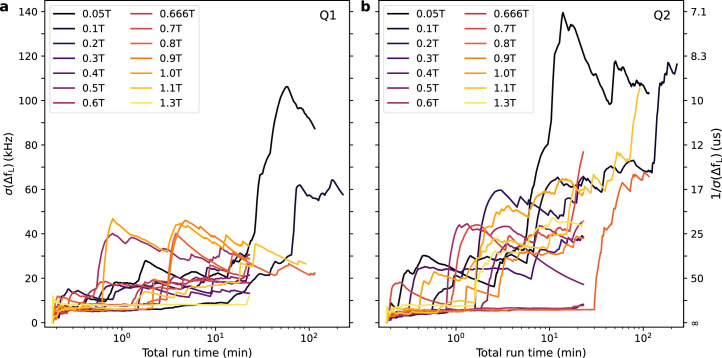
Larmor frequency variation as function of *B*_0_. **a**,**b**, Standard deviation of the Larmor frequency variation *σ*(Δ*f*_L_) obtained from real-time Larmor frequency feedback during interleaved Ramsey measurements as a function of total run time for a range of magnetic field amplitudes *B*_0_ of qubit 1 and 2, respectively. The second y-axis on the right shows the inverse 1/*σ*(Δ*f*_L_) as an approximation for expected ${T}_{2}^{* }$.

Third, sequences between which the Larmor frequency feedback exhibits a large frequency change can be removed from the GST dataset, but this has a significant effect only on the stochastic IZ error generator of the XI gate (Extended Data Fig. 7). Moreover, the spread of the Larmor frequency is independent of *B*_0_, whereas the Stark shift increases linearly (Extended Data Figs. 8 and 9) and the spin–orbit interaction in qubit 1 is stronger compared with qubit 2, as can be seen in Fig. 3a,b. The standard deviation of the Larmor frequency feedback increases in a step-like manner as a function of total experiment runtime for distinct instances across all magnetic fields (Extended Data Fig. 10). Hence, these slow timescale jumps probably originate from qubit 2 coupling strongly to the nuclear spin states of the residual ^29^Si atoms. As such, they contribute significantly to the gate infidelities and are the cause of the above-described observations^2,42,43^. These slow jumps in the Larmor frequency do not contribute to ${T}_{2}^{{\rm{Hahn}}}$ and, therefore, do not contradict ${T}_{2,{\rm{Q}}2}^{{\rm{Hahn}}} > {T}_{2,{\rm{Q}}1}^{{\rm{Hahn}}}$.

**Fig. 5:**
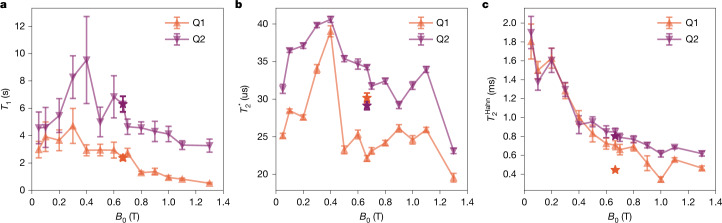
Magnetic field dependence of coherence times for device A. **a**–**c**, Spin lifetime *T*_1_ (**a**), Ramsey ${T}_{2}^{* }$ (**b**) and Hahn echo ${T}_{2}^{{\rm{Hahn}}}$ (**c**) metrics as functions of the applied magnetic field amplitude *B*_0_. We measured both qubits for each experiment interleaved and repeated this 50 (*T*_1_), 10,000 (${T}_{2}^{* }$) and 2,000 (${T}_{2}^{{\rm{Hahn}}}$) times. Ramsey experiments at *B*_0_ = 0.05 T, 0.1 T, 0.8 T and 1.1 T were repeated 50,000, 100,000, 50,000 and 40,000 times. The Hahn echo experiment at *B*_0_ = 1.3 T was repeated 500 times. Between each repeat we used real-time Larmor frequency feedback following the protocol described in ref. ^39^. The data were fitted using the functions described in Extended Data Fig. 2. Data shown as stars correspond to metrics measured during two-qubit benchmarking (Extended Data Fig. 2), using a method and gate voltages that differed from those used for the magnetic field sweep. Error bars represent the 95% confidence level.

**Extended Data Fig. 11:**
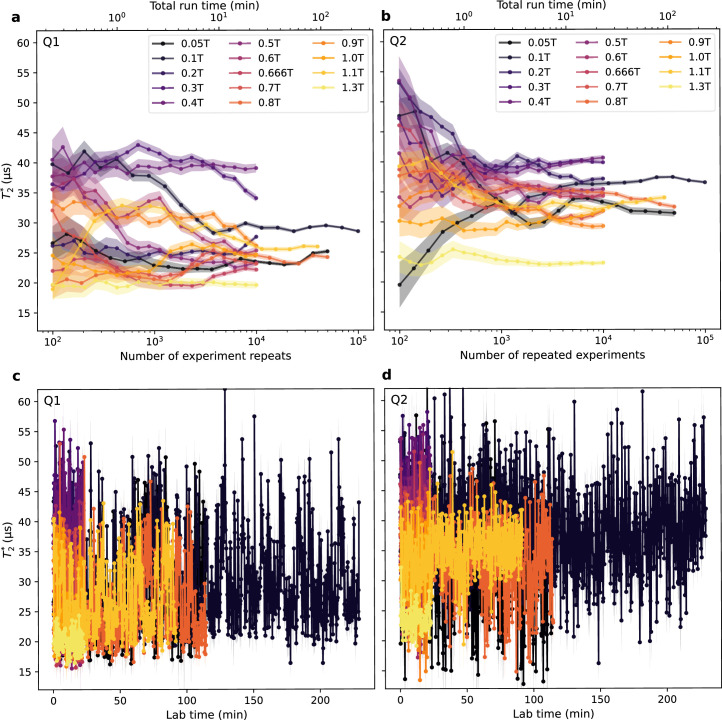
Cumulative ${T}_{2}^{* }$ from repeated Ramsey experiments as function of *B*_0_. **a**,**b**, Cumulative ${T}_{2}^{* }$ of qubit 1 and 2 as a function of increasing number of experiment repeats and total measurement time for a range of magnetic field amplitudes *B*_0_, respectively. Each experiment is defined by 101 shots of the Ramsey circuit with varying wait time. **c**,**d**, ${T}_{2}^{* }$ of qubit 1 and 2 as a function of lab time, respectively. Each data point is obtained by 100 repeated Ramsey experiments taking a total of approximately 15 s. We use real-time Larmor frequency feedback between Ramsey experiment repeats. Shaded error regions represent the 95% confidence level.

In terms of qubit device performance, Fig. 5 shows spin lifetime and coherence metrics as functions of the magnetic field *B*_0_. *T*_1_ and ${T}_{2}^{* }$ first increase up to *B*_0_ = 0.3–0.4 T, followed by a decrease for larger *B*_0_. Because ${T}_{2}^{* }$ is sensitive to d.c. noise, it samples 1/*f* noise down to frequencies corresponding to the total time of the experiment, which renders it a bad metric for comparing different qubits or qubit platforms. Including Larmor frequency feedback in the experiment reduces the contribution of low-frequency 1/*f* noise^44^. We demonstrate this in Extended Data Fig. 11a,b by plotting ${T}_{2}^{* }$ as a function of the cumulative measurement time. Most curves are approximately flat, showing the effectiveness of the feedback mechanism. Extended Data Fig. 11c,d show the variation of ${T}_{2}^{* }$ as a function of laboratory time.

The longest measured coherence times for device A include Hahn echo coherence time ${T}_{2}^{{\rm{Hahn}}}=\text{1,900}\,{\rm{\mu }}{\rm{s}}$ (1,805 μs) for qubit 2 (qubit 1) measured at the low magnetic field of *B*_0_ = 50 mT, as well as ${T}_{2}^{* }$ = 40.6 μs (39.0 μs) and *T*_1_ = 9.5 s (4.7 s). The long ${T}_{2}^{{\rm{Hahn}}}$ and its small variation between both qubits indicate that magnetic noise dominates at low fields. On increasing *B*_0_, the Stark shift increases linearly (Extended Data Fig. 9), with steeper slopes for qubit 1 (Δ*Ω*_Stark_(P1, J, P2) = (−19.9(4), 31.3(3), 14.2(4)) MHz V^−1^ T^−1^) than for qubit 2 ((0, 3.2(2), 16.7(4)) MHz V^−1^ T^−1^), manifesting in stronger coupling to charge noise through the spin–orbit interaction and resulting in reduced coherence, with a 33% larger ${T}_{2,Q2}^{{\rm{Hahn}}}=617(26)\,{\rm{\mu }}{\rm{s}}$ (${T}_{2,Q1}^{{\rm{Hahn}}}=464(22)\,{\rm{\mu }}{\rm{s}}$) at *B*_0_ = 1.3 T. The observed Stark shift is entirely caused by the intrinsic spin–orbit coupling of silicon electrons confined at the Si/SiO_2_ interface. For device A, the external magnetic field points in the [110] lattice orientation, where the variability of the *g* factor is highest and the Dresselhaus spin–orbit component dominates. This type of spin–orbit coupling is largely influenced by atomistic roughness at the Si/SiO_2_ interface^29^. In the presence of a gate bias, the quantum dot moves under the rough interface, shifting its *g* factor. The magnitude and sign of the resulting Stark shift depends on the direction in which the gate moves the quantum dot. Typical values range between −80 MHz V^−1^ T^−1^ to 80 MHz V^−1^ T^−1^ (ref. ^25^). In device A, we obtained −20 MHz V^−1^ T^−1^ to 30 MHz V^−1^ T^−1^, which falls within that range. The magnetic field dynamics of the spin coherence times are distinct from previous results for academic devices^7^, and the exact physical origin requires a more detailed study, which is beyond the scope of this work.

Looking forward, the increase in ${T}_{2}^{{\rm{Hahn}}}$ for low fields is extremely encouraging because it indicates that the qubits are amenable to more scalable control techniques. Such approaches continuously drive the spin by using a modulated microwave field to decouple it from noise and eliminate free precession (${T}_{2}^{{\rm{Hahn}}}\gg {T}_{2}^{* }$)^36,45^. Operation at lower fields requires a different modality of the two-qubit gate. To achieve similarly fast gates, a SWAP-like operation will be implemented. Furthermore, at low fields an always-on, global control is beneficial for avoiding idling errors and using the long decoupling times ${T}_{2}^{{\rm{Hahn}}}\gg {T}_{2}^{* }$ observed. This can be achieved by aligning the magnetic field direction such that the Larmor frequencies of the qubits become degenerate^45^. The key challenge for that operation modality is sufficiently large Stark shifts.

Our chosen implementation of an entangling gate (CZ) is faster than decoupling schemes, but it relies on the exchange interaction being smaller than the Zeeman energy difference of the two qubits (*J*_exc_ ≪ Δ*E*_Z_). We chose *B*_0_ values in the range 0.662–0.7 T to meet this requirement, but in future, we intend to explore other two-qubit gate implementations^8^.

## Conclusions

We have demonstrated high-fidelity control of semiconductor qubits industrially manufactured in a 300-mm pilot line. In contrast to previous publications in which charge noise is identified as the key limiting factor^3–5,8,38,46,47^, our results are strongly encouraging from a noise-analysis perspective, both in terms of the fidelity values themselves, and in view of the clear pathway they pave towards further improvement through further nuclear purification. Further isotopic enrichment of the 400-ppm ^29^Si used in this work to levels at or below 50 ppm has already been demonstrated in academic prototype devices^7^. This is consistent with previous results showing the effect of nuclear spin noise on coherence times in Si/SiGe quantum-dot spin qubits^20^.

We have shown that using engineering practices in an industrial setting can lead to state-of-the-art spin-qubit performance when coupled with a systematic search for target performance metrics. It is crucial to understand the connection between qubit performance and measurements that can be performed during the fabrication process, such as electrical noise and Hall bar transport^19,48^. These investigations are in their infancy and will require an increasingly accurate model of the quantum behaviour of electrons in semiconductor devices.

In the long term, further improvements will be required on the qubit front, for which our noise analysis provides a first-order angle of attack. To reduce the overhead for fault-tolerant quantum computing^32^, 99.9% fidelities across all operations is a realistic target.

At this stage, a statistical analysis of these qubits at a larger scale is not yet available. The consistent calibration of qubits at the level demonstrated here remains an intensive manual process with a number of scientific insights gained along the way. Automation of this process needs to mature to consistently achieve these results in a mass-characterization campaign^49^. Having silicon–metal–oxide–semiconductor (SiMOS) qubits fabricated in a foundry environment readily available will facilitate the future demonstration of large-scale SiMOS qubit arrays. This has so far been challenging for SiMOS spin qubits because it has been difficult to fabricate devices with small gate pitches and low charge-noise interfaces.

These results could be leveraged to design quantum error correction strategies to cater to the specific demands of spin qubits. However, further studies will be needed to characterize qubit operations in a more scalable setting, such as under a single global microwave field and under elevated temperatures, which will inevitably be imposed by the heat dissipated by the co-integrated control electronics on the CMOS chip^50–52^. Our reproducible demonstrations of high-quality qubit operation made using 300-mm foundry processes offer a proof of principle that should encourage further industrial research into far more complex technologies that are at the disposal of foundries.

## Methods

### Fabrication

**Extended Data Fig. 12:**
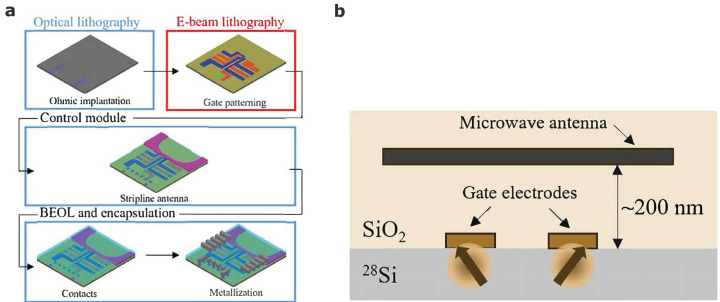
Device fabrication and schematic. **a**, Fabrication flow schematic. **b**, Schematic depicting the location of the microwave antenna with respect to the quantum dot location. Panel **a** adapted with permission from ref. ^59^, IEEE (The Japan Society of Applied Physics, 2023).

The fabrication method used at imec was optimized for low charge noise, high uniformity^19,31,53,54^ and qubit-specific integration with a gate pitch smaller than 100 nm. Fast fabrication cycles and design flexibility were ensured by optimally combining optical lithography and electron-beam lithography. Fabrication started with the epitaxial growth of an isotopically enriched layer of silicon with a residual concentration of 400 ppm ^29^Si. A high-quality thermally grown oxide forms the Si/SiO_2_ interface at which the charge of the electron spin qubit accumulates. Dedicated cleaning processes and 300-mm pilot line process control are key to the development of the high-quality interface^19^. Next, a triple-layer overlapping polysilicon gate stack was formed using electron-beam lithography and dry etching, with each gate separated by a thin interstitial high-temperature oxide deposit^19^. The highly doped polysilicon gates reduced the interface strain at cryogenic temperatures^54,55^. Finally, an aluminium strip line was patterned so that single spin states could be manipulated using electron spin resonance, offering a control solution at the few-qubit level^50,52^ (Extended Data Fig. 12).

### Measurement

Devices A, B, C and D were measured in Bluefors XLD400, Bluefors LD400, and two different Oxford Instruments K100 Kelvinox dilution refrigerators, respectively, and mounted on the cold finger. Previous work conducted with similar experimental set-ups indicates that the electron temperature was 200–300 mK (ref. ^56^). An American Magnetics AMI430 supplied static external magnetic fields to devices A, B and C, with the field for A pointing in the [110] direction of the Si lattice and those for B and C pointing in the [1$\overline{1}$0] direction. The field for device D was supplied by an Oxford Instruments IPS120-10 pointing in the [1$\overline{1}$0] direction of the Si lattice.

We used a Q-Devil QDAC-II to supply d.c. voltages through filtered lines with a bandwidth of up to 20 Hz. Dynamic voltage pulses were generated with a field-programmable gate array, in this experiment the Quantum Machines Operator-X+ (OPX+). The pulses were combined with d.c. biases using custom linear bias combiners at room temperature. The OPX+ had a sampling time of 1 ns.

The dynamic pulse lines in the fridge had a bandwidth of up to 50 MHz, which translated into a minimum rise time of 20 ns. We accounted for this by smoothing out fast a.c. pulsing. Microwave pulses were generated by a vector signal generator (PSG8267D, Keysight). The in-phase and quadrature and pulse modulation waveforms were generated by the OPX+.

The charge sensor comprised a single-island SET in d.c. mode. The SET current integrated for *t*_int_ = 100 μs was amplified using a room-temperature *I*–*V* converter (Basel SP983c) and sampled by the OPX+. At this stage, we used d.c. mode read-out, which was limited by the bandwidth and noise of the room-temperature transimpedance amplifier, leaving high-bandwidth cryogenic current amplification for future work.

Gate times were optimized based on a trade-off between speed and lifetime. The magnetic field amplitude *B*_0_ was chosen in the range 0.662–0.7 T, such that the single-qubit Rabi *Q*_Rabi_ = *f*_Rabi_*T*_Rabi_ was maximized. Simultaneously, the chosen implementation of entangling gate relied on the exchange interaction being smaller than the Zeeman energy difference of the two qubits (*J*_exc_ ≪ Δ*E*_Z_). We chose our *B*_0_ values to meet this requirement.

## Online content

Any methods, additional references, Nature Portfolio reporting summaries, source data, extended data, supplementary information, acknowledgements, peer review information; details of author contributions and competing interests; and statements of data and code availability are available at 10.1038/s41586-025-09531-9.

## Data Availability

The data supporting this work are available at Zenodo (10.5281/zenodo.15571656)^57^.
